# Long noncoding RNA HOTAIR regulates the stemness of breast cancer cells *via* activation of the NF-κB signaling pathway

**DOI:** 10.1016/j.jbc.2022.102630

**Published:** 2022-10-20

**Authors:** Jiajia Wang, Xingzhu Liu, Ping Li, Junrong Wang, Yu Shu, Xinyu Zhong, Zhen Gao, Jingyi Yang, Yashuang Jiang, Xile Zhou, Geng Yang

**Affiliations:** 1 Department of Clinical Medicine & Key Laboratory of Novel Targets and Drug Study for Neural Repair of Zhejiang Province, School of Medicine, Zhejiang University City College, Hangzhou, China; 2 Core Facilities, School of Medicine, Zhejiang University, Hangzhou, China; 3 School of Bioengineering, Hangzhou Medical College, Hangzhou, China; 4 College of Pharmacy, Zhejiang University of Technology, Hangzhou, China; 5 Department of Colorectal Surgery, The First Affiliated Hospital, Zhejiang University, Hangzhou, China

**Keywords:** LncRNA, cancer stem cell, NF-κB signaling, IκBα, BCSC, breast cancer stem cell, CSC, cancer stem cell, EZH2, enhancer of zeste homolog 2, H3K27me3, histone H3 lysine 27 trimethylation, HOTAIR, Hox transcript antisense intergenic RNA, lncRNA, long noncoding RNA, NF-κB, nuclear factor-kappa B, PRC2, polycomb repressive complex 2, SUZ12, suppressor of zeste 12

## Abstract

Breast cancer is the most prevalent cancer among women, and it is characterized by a high rate of tumor development and heterogeneity. Breast cancer stem cells (CSCs) may well contribute to these pathological properties, but the mechanisms underlying their self-renewal and maintenance are still elusive. Here, we found that the long noncoding RNA HOTAIR is highly expressed in breast CSCs. HOTAIR is required for breast CSC self-renewal and tumor propagation. Mechanistically, we demonstrate that HOTAIR recruits the PRC2 protein complex to the promoter of IκBα to inhibit its expression, leading to activation of the NF-κB signaling pathway. The activated NF-κB signaling promotes downstream c-Myc and Cyclin D1 expression. Furthermore, our analysis of clinical samples from the GEPIA database indicated that the IκBα level, as well as the survival rate of patients, with high HOTAIR expression was significantly lower than that of patients with relatively low HOTAIR expression. Our data suggest that HOTAIR-mediated NF-κB signaling primes breast CSC self-renewal and tumor propagation. In sum, we have identified HOTAIR-based NF-κB signaling regulatory circuit that promotes tumorigenic activity in breast CSCs, further indicating that HOTAIR could be a promising target for clinical treatment of breast cancers.

Breast cancer is the most common disease among women in the world (1) and accounts for 12.2% of new cases and 9.6% of cancer deaths in China (2). Although multiple therapeutics are available, their efficacy needs to be further improved, especially for breast cancers which usually harbor distinct mutations on estrogen receptor, progesterone receptor, and Her2 (3, 4, 5, 6). Recent studies have suggested that heterogeneity is a result of the hierarchical organization of tumor cells by a subset of cells with stem/progenitor cell features known as cancer stem cells (CSCs) (7). It has gradually been confirmed that the reason why breast cancer is so difficult to cure is due to the existence of breast cancer stem cells (BCSCs), which mediate tumor development, chemotherapy resistance, and cancer recurrence (8, 9). Growing evidence has also shown that different signaling pathways play important roles in the initiation and maintenance of BCSCs. For example, Pygo2 stimulated MDR1 expression in the resistant cells *via* the Wnt/β-catenin pathway and the downregulation of Pygo2 restored the chemotherapeutic drug sensitivity of the resistant cells and decreased the BCSC population in these cells in response to chemotherapy (10). The PAK1-STAT3 signaling pathway promoted IL-6 gene transcription and BCSCs formation (11). Although various cell signaling pathways have been reported to be involved in the regulation of BCSCs, how breast CSCs sustain their self-renewal remains largely unknown.

Long noncoding RNAs (lncRNAs) are defined as transcripts longer than 200 nucleotides (nt) with limited coding potential and play roles in a wide range of biological processes by regulating gene expression in cis or in trans through diverse mechanisms (12, 13, 14). LncRNA-mediated biology has been implicated in many cellular processes (14) and is usually abnormally expressed in cancer, which have also been reported to act as a prominent layer of transcriptional regulation often by collaborating with chromatin remodeling complexes (15, 16, 17). HOTAIR (Hox transcript antisense intergenic RNA) is one of these lncRNAs transcribed from the HOXC locus and is able to reprogram chromatin state to promote cancer metastasis in breast cancers at its first discovery (15). Subsequently, HOTAIR has been associated with the metastasis of many types of cancers, including colorectal (18), pancreatic (19), lung (20), and breast cancers (21). Furthermore, HOTAIR has also been associated with poor prognosis and advanced tumor stage in cancers (22, 23, 24). Its prometastatic function is attributed to HOTAIR-induced epigenetic silencing of metastasis-suppressor–genes and reorientation of the polycomb repressive complex 2 (PRC2) (15, 25). However, the role of HOTAIR in CSCs, especially in BCSCs, are not well understood.

In this study, we found that HOTAIR was more highly expressed in BCSCs than in non-CSCs. HOTAIR was required for the self-renewal maintenance of BCSCs, and tumorigenic capacity of BCSCs was enhanced while HOTAIR was overexpressed. Mechanically, HOTAIR recruited the PRC2 to inhibit IκBα expression, leading to activation of nuclear factor-kappa B (NF-κB) signaling for priming breast CSC self-renewal.

## Results

**Figure 1:**
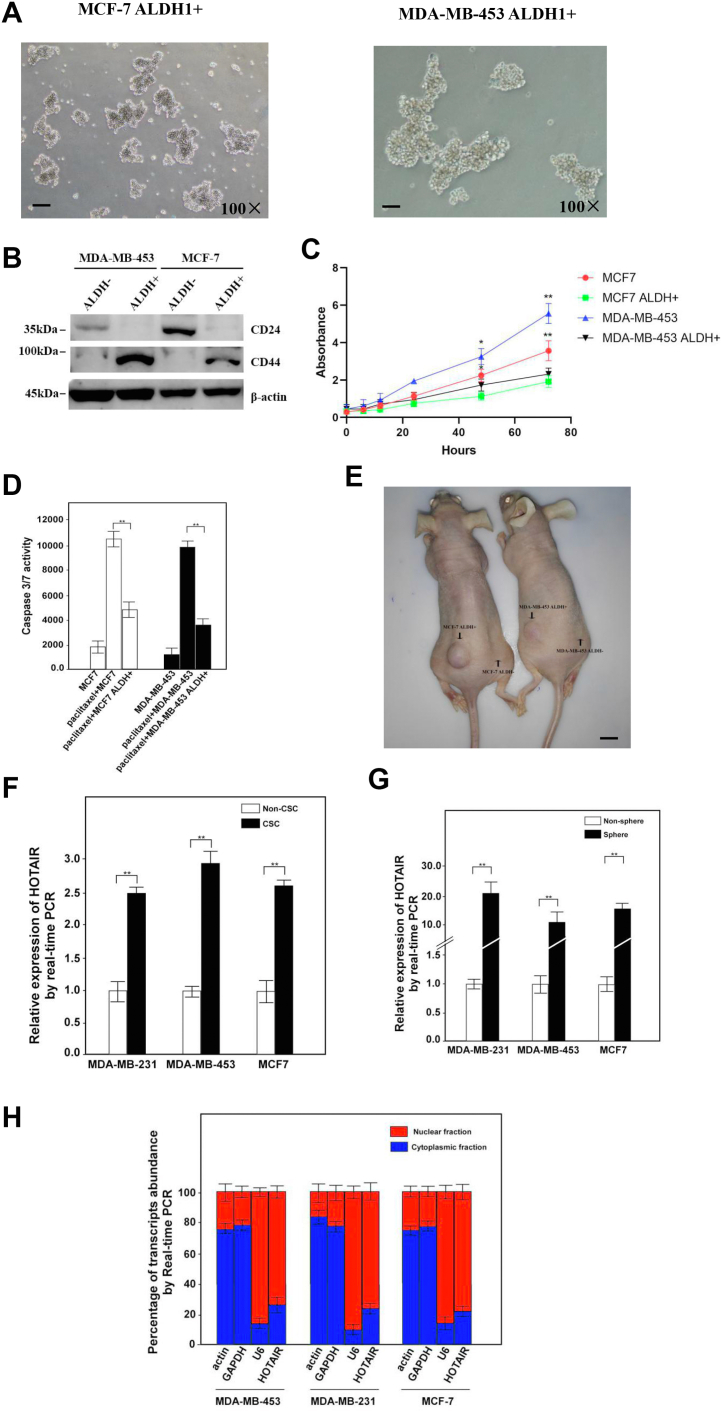
**LncRNA HOTAIR is highly expressed in breast CSCs.***A*, MCF-7 ALDH1+ and MDA-MB-453 ALDH1+ cells sorted by FACS were cultured in medium and then examined under microscope. Cells were magnified by 100 folds and the scale bar represented 10 μm. *B*, CD44 and CD24 were detected by Western blotting in the indicated cells. β-actin was used as an internal control. *C*, the growth rate of the ALDH+ or ALDH- cells was measured by MTT. Results were shown as means ± SD. *D*, the Caspase 3/7 activity was measured with or without paclitaxel (1 μM) treatments in the indicated cells. Results were shown as means ± SD. *E*, ALDH+ or ALDH- breast cancer cells were transplanted into the subcutaneous area of the *left* or *right* limb of the nude mouse and the tumors were examined 1 month after the injection. The number of mice employed in this experiment was ten and the scale bar represented 1 cm. *F*, breast CSCs (ALDH+) and non-CSCs (ALDH-) sorted from the corresponding cell lines by FACS were subjected to the analysis of HOTAIR by real-time PCR. Relative gene expression was normalized to endogenous β-actin. Results are shown as means ± SD. *G*, HOTAIR was analyzed in oncosphere and non-oncosphere cells derived from three breast cell lines. Results are shown as means ± SD. *H*, breast CSCs were fractionated followed by quantitative real-time PCR for the detection of the corresponding transcripts. β-actin and GAPDH were served as positive controls for cytoplasmic gene expression. U6 RNA was served as a positive control for nuclear gene expression. Data are representative of at least three independent experiments. ∗*p* < 0.05, ∗∗*p* < 0.01 by two-tailed Student’s *t* test. CSC, cancer stem cell; HOTAIR, Hox transcript antisense intergenic RNA.

Although HOTAIR has been found to play the role of proto-oncogene in different types of cancer, the function of HOTAIR in stem cells has not been effectively studied, especially in BCSCs. In order to study the function of HOTAIR in BCSCs, we first prepared to isolate the corresponding cancer stem cells from MCF-7 and MDA-MB-453. ALDH1 was selected as a target for the isolation of BCSCs by FACS. 1.6% and 1.2% of MDA-MB-453 and MCF-7 cells in the side population were sorted as ALDH1-positive cells, respectively (Fig. S1, *A* and *B*), which was then cultured in the stem cell medium. The results showed that ALDH1+ cells could grow in suspension and have the ability of forming oncosphere while the ALDH- cells could not grow in the same culture medium which was probably due to the lack of stemness in ALDH- cells (Fig. 1*A*). The detection of CD44/CD24 in sorted cells showed that the level of CD44 was significantly upregulated, while CD24 was dramatically downregulated in ALDH1+ cells (Fig. 1*B*), which was in line with the expression profiles of CD44+/CD24- in CSCs. The growth rate of ALDH1+ cells was also significantly lower than that of control cells (Fig. 1*C*). Moreover, the induction of apoptosis in ALDH+ cells was also lower than that of ALDH- cells (Fig. 1*D*) while treated with paclitaxel. The transplantation of 10^2^ cells of ALDH+ cells or ALDH- cells into the subcutaneous area of the left or right limb of the nude mouse resulted in the formation of tumor only in the area where ALDH+ cells were injected (Fig. 1*E*). Based on these results, we identified ALDH+ cells from MCF7 and MDA-MB-435 as BCSCs. Meanwhile, MDA-MB-231CSC was obtained as a gift. Then, we first examined the expression of HOTAIR in the sorted CSCs immediately from FACS as well as in the cultured oncosphere CSCs. Both the results showed that ALDH+ cells or cultured CSCs had significantly higher expression of HOTAIR than that of ALDH- cells (Fig. 1, *F* and *G*). We then analyzed the distribution of HOTAIR in these stem cells and found that HOTAIR was mainly localized in the nucleus (Fig. 1*H*), indicating that HOTAIR might play its role in the nucleus. In summary, these data indicate that HOTAIR was highly expressed in BCSCs.

**Figure 2:**
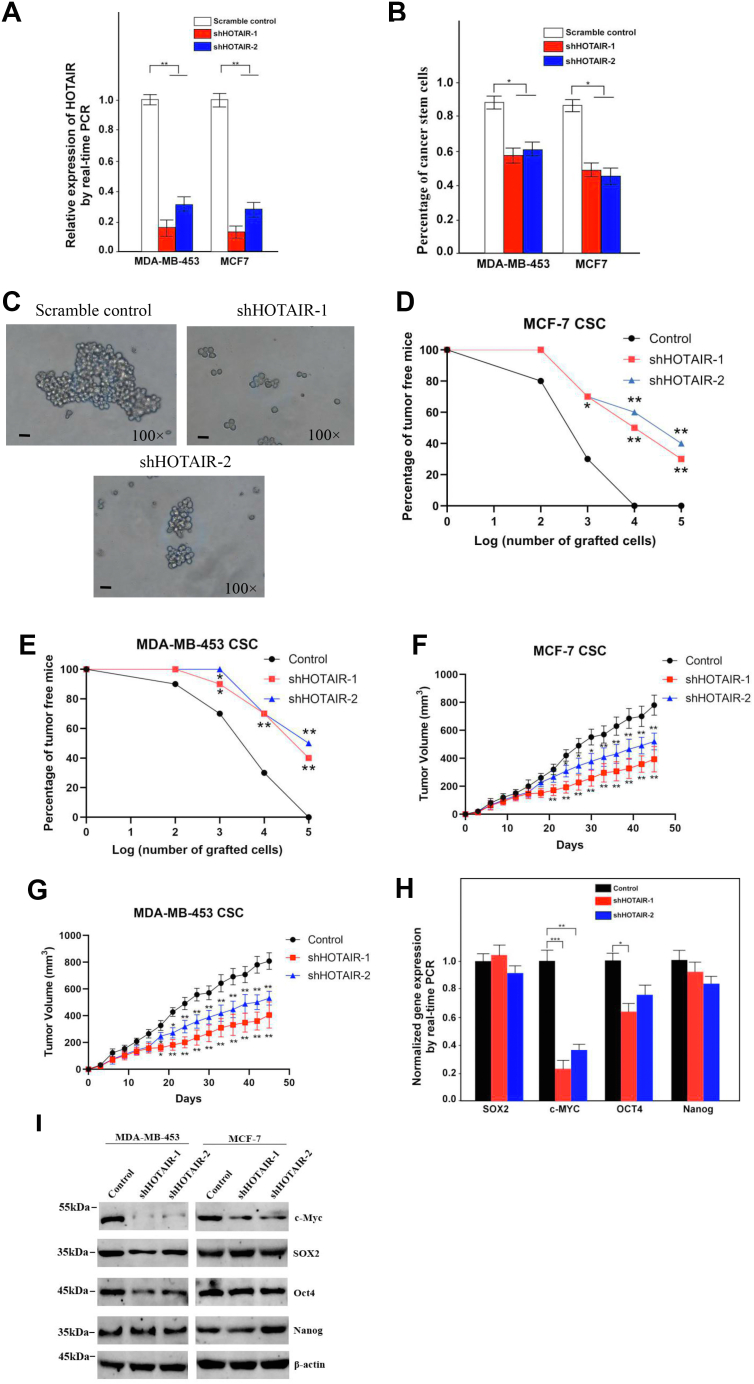
**HOTAIR is required for the self-renewal maintenance of breast CSCs.***A*, HOTAIR was silenced in breast CSCs by two independent shRNAs and analyzed by RT-PCR. HOTAIR-silenced stable cell lines were established. Data are shown as means ± SD. *B*, ALDH1+ (CSC) subpopulations were detected in HOTAIR-depleted cells by FACS analysis. Results are shown as means ± SD. *C*, HOTAIR depletion causes a diminished oncosphere-forming capacity in MCF7 CSC. Cells were magnified by 100 folds and the scale bar represented 10 μm. *D*, different numbers of HOTAIR-silenced or control breast CSC of MCF7 were diluted and subcutaneously implanted into NOD/SCID nude mice. Tumor-free mice were measured 1 month after injection. N = 10 for each group. *E*, different numbers of HOTAIR-silenced or control breast CSC of MDA-MB-453 were diluted and subcutaneously implanted into NOD/SCID nude mice. Tumor-free mice were measured 1 month after injection. N = 10 for each group. *F*, HOTAIR-silenced or control breast CSC of MCF7 were diluted and subcutaneously implanted into NOD/SCID nude mice. Tumors were observed over 1 month. N = 10 for each group. *G*, HOTAIR-silenced or control breast CSC of MDA-MB-453 were diluted and subcutaneously implanted into NOD/SCID nude mice. Tumors were observed over 1 month. N = 10 for each group. *H*, pluripotent transcription factors were analyzed in HOTAIR-depleted cells by quantitative real-time PCR. Results are shown as means ± SD in MDA-MB-453. *I*, the level of pluripotent transcription factors were analyzed in HOTAIR-depleted cell by Western blotting. β-actin was used as a control. CSC, cancer stem cell; HOTAIR, Hox transcript antisense intergenic RNA (∗ indicates *p* < 0.05; ∗∗ indicates *p* < 0.01; ∗∗∗ indicates *p* < 0.001.).

To determine the function of HOTAIR in self-renewal of BCSCs, two shRNAs expressed by lentivirus against HOTAIR were employed and shHOTAIR-1 achieved more effective knockdown efficiency (Fig. 2*A*). The result showed that HOTAIR knockdown remarkably impaired generation of the fraction of ALDH+ cells (CSCs) (Fig. 2*B*). Importantly, HOTAIR depletion also dramatically reduced oncosphere formation of MCF7 CSC (Fig. 2*C*). The role of HOTAIR in tumor-initiating formation was next explored. We performed subcutaneous injection of NOD/SCID nude mice with CSCs stably knockdown of HOTAIR or the control cells. The results showed HOTAIR depletion resulted in a much weaker tumor presence compared with control cells as assessed by a limiting dilution xenograft analysis (Fig. 2, *D* and *E*), suggesting that HOTAIR knockdown reduced tumor-initiating capacity. Moreover, HOTAIR depletion significantly suppressed xenograft tumor growth as examined by tumor volume for 4 months (Fig. 2, *F* and *G*). We next aimed to get a preliminary understanding of how HOTAIR participated in the stem cell signaling pathway, so the levels of Sox2, Nanog, Oct4, and c-Myc were examined upon knockdown of HOTAIR. Notably, HOTAIR depletion significantly reduced the expression of pluripotent transcription factors c-Myc compared with scrambled control (Fig. 2*H*). By contrast, HOTAIR knockdown did not dramatically affect OCT4, Nanog expression, which was further confirmed by the detection of protein levels (Fig. 2*I*). Unexpectedly, we observed there was an OCT4 reduction in MDA-MB-453 but not in MCF7 after HOTIAR knockdown for the reasons we did not quite understand yet. Overall, these data indicate HOTAIR silencing abrogates the tumorigenic capacity of BCSCs.

**Figure 3:**
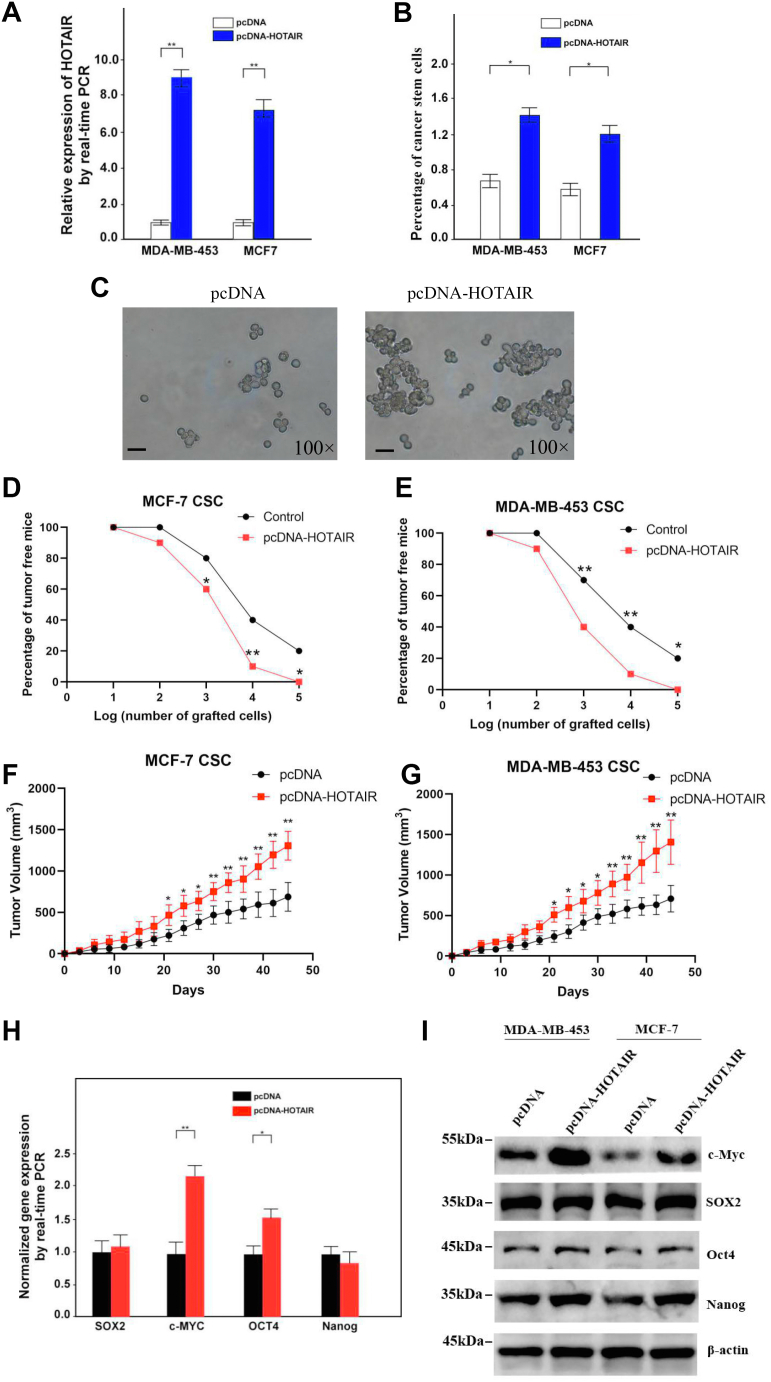
**Tumorigenic capacity of breast CSCs is enhanced by HOTAIR overexpression.***A*, HOTAIR overexpressing breast tumor cells were established. Data are shown as means ± SD. *B*, ALDH1+ (CSC) subpopulations were detected in HOTAIR overexpressed cells by FACS analysis. Results are shown as means ± SD. *C*, HOTAIR overexpression causes an increased oncosphere-forming capacity in breast cancer stem cells. Cells were magnified by 100 folds and the scale bar represented 10 μm. *D*, different numbers of HOTAIR-overexpressing or control breast CSC of MCF7 were diluted and subcutaneously implanted into NOD/SCID nude mice. Tumor-free mice were measured 1 month after injection. N = 10 for each group. *E*, different numbers of HOTAIR-overexpressing or control breast CSC of MDA-MB-453 were diluted and subcutaneously implanted into NOD/SCID nude mice. Tumor-free mice were measured 1 month after injection. N = 10 for each group. *F*, HOTAIR overexpressing or control breast CSC of MCF7 were diluted and subcutaneously implanted into NOD/SCID nude mice. Tumors were observed over 1 month. N = 10 for each group. *G*, HOTAIR overexpressing or control breast CSC of MDA-MB-453 were diluted and subcutaneously implanted into NOD/SCID nude mice. Tumors were observed over 1 month. N = 10 for each group. *H*, overexpression of HOTAIR resulted in elevated expression of pluripotent factors in breast cells as assessed by quantitative real-time PCR. Data are shown as means ± SD. *I*, the expression of pluripotent factors were determined by Western blot after HOTAIR overexpression. β-actin was used as a control. CSC, cancer stem cell; HOTAIR, Hox transcript antisense intergenic RNA (∗ indicates *p* < 0.05; ∗∗ indicates *p* < 0.01.).

We next overexpressed HOTAIR in MCF7 and MDA-MB-453 cell lines and established HOTAIR stably overexpressing cell lines (Fig. 3*A*), which were then used for the sorting of ALDH+ cells. Notably, HOTAIR overexpression significantly raised the percentage of ALDH+ cells, indicating that HOTAIR overexpression increased the portion of CSCs in MCF7 and MDA-MB-453 (Fig. 3*B*). HOTAIR overexpression also significantly enhanced oncosphere formation of the CSCs (Fig. 3*C*). Tumor-initiating formation was then determined by a limiting dilution injection of these CSCs into NOD/SCID nude mice, and the result showed that HOTAIR overexpression resulted in a much stronger tumor presence (Fig. 3, *D* and *E*). Moreover, HOTAIR overexpression dramatically enhanced xenograft tumor growth (Fig. 3, *F* and *G*). Furthermore, we also determined the expression of pluripotent transcription factors Sox2, Nanog, Oct4, and c-Myc in HOTAIR-overexpressed cells and found that only c-Myc but not other three factor was upregulated on both mRNA and protein level (Fig. 3, *H* and *I*). Taken together, these data indicate that HOTAIR plays a critical role in the self-renewal maintenance of BCSCs.

**Figure 4:**
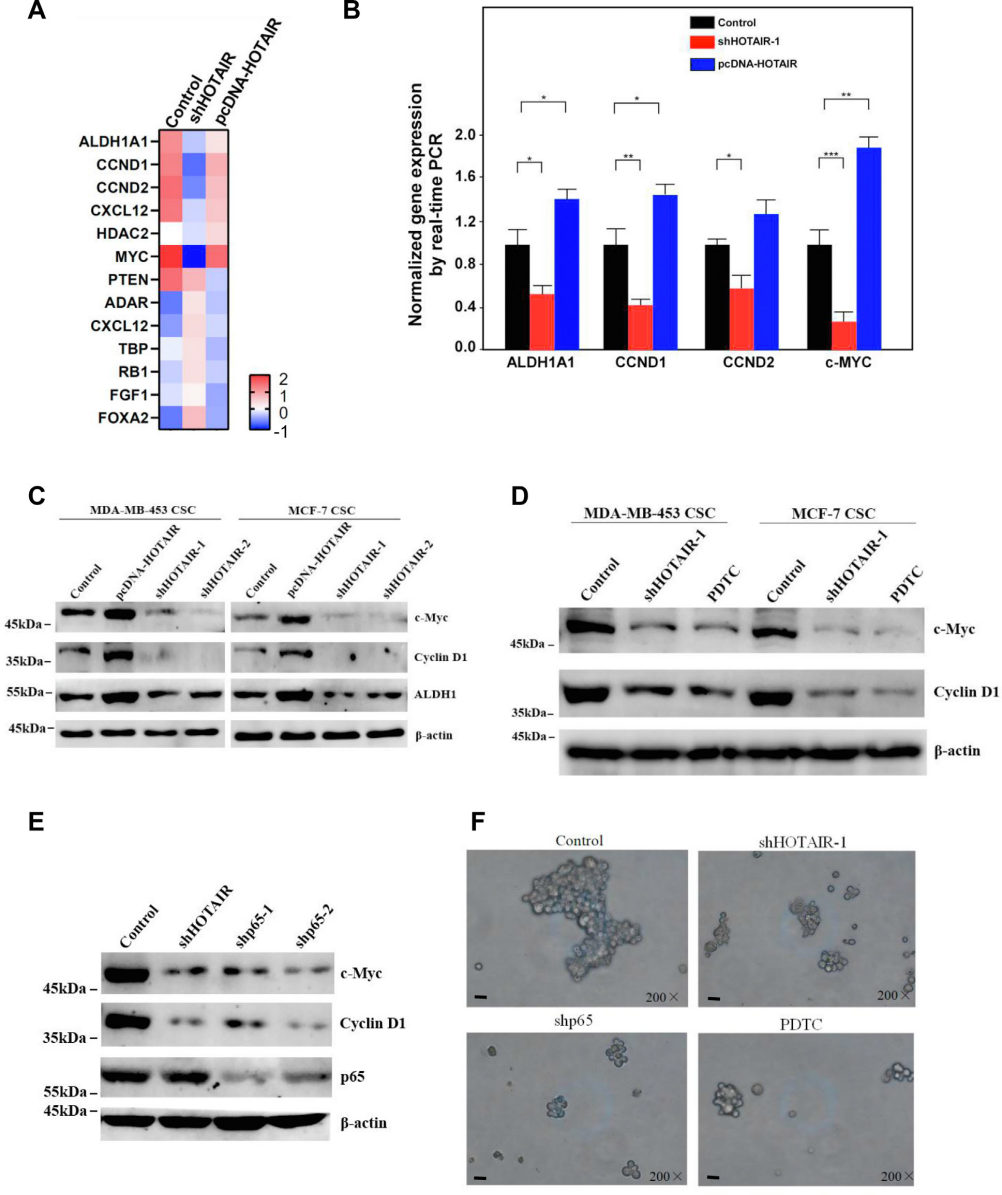

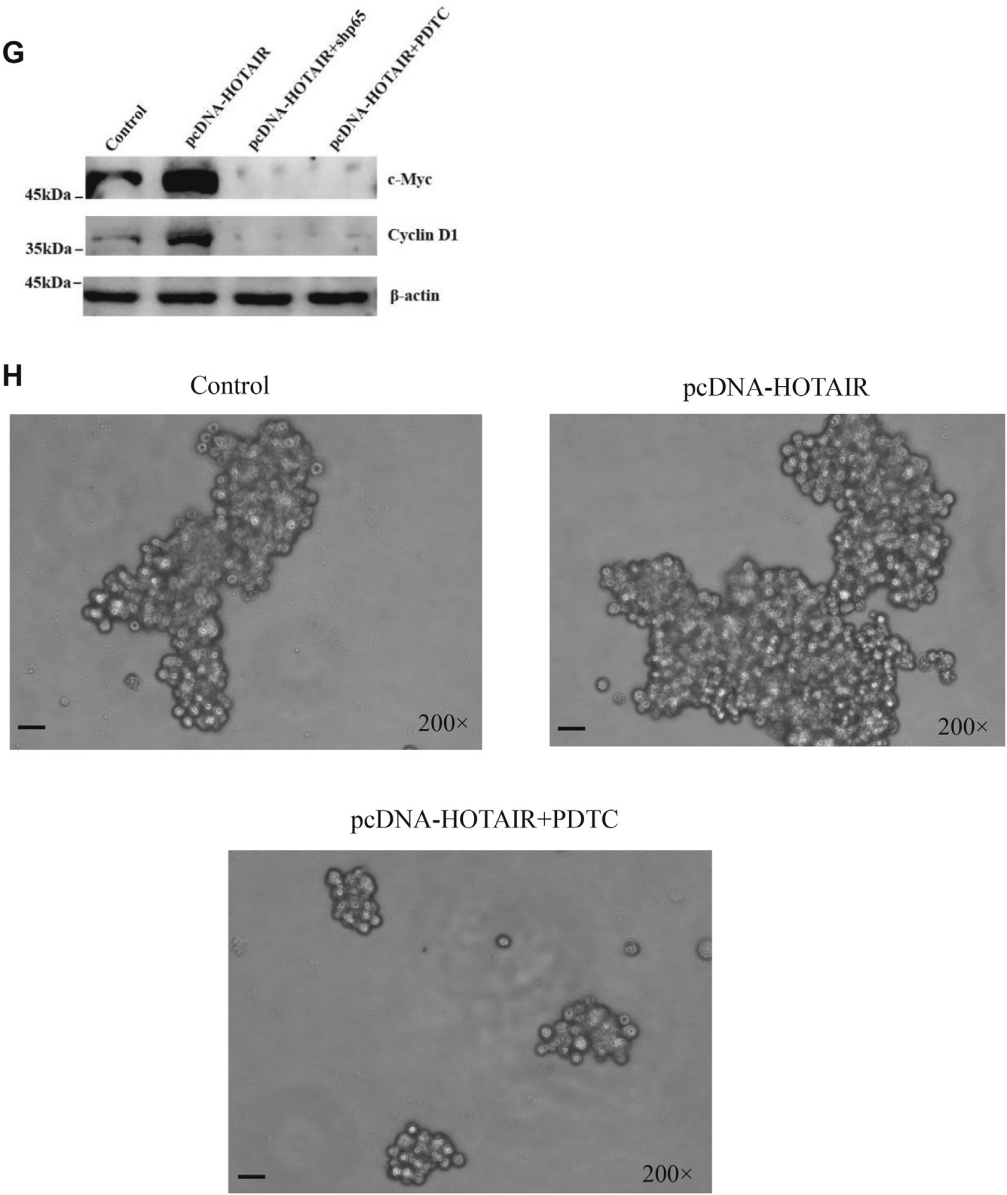
**HOTAIR regulates the stemness of breast cancer cells through NF-κB signaling pathway.***A*, heat map from mRNA real-time PCR data. Thirteen RNA transcripts (*p* < 0.05) from MCF7 CSC with HOTAIR overexpression or knockdown were differently expressed in the indicated treatments. *B*, the indicated genes were subjected to the expression analysis by real-time PCR. Relative gene expression was normalized to endogenous β-actin. Results are shown as means±SD. *C*, the expression of indicated genes were determined by Western blot after HOTAIR overexpression or knockdown. β-actin was used as an internal control. *D*, the expression of indicated genes were determined by Western blot after HOTAIR knockdown or PDTC (50 μM) treatment. β-actin was used as an internal control. *E*, the expression of indicated genes were determined by Western blot after HOTAIR knockdown or p65 knockdown. β-actin was used as an internal control. *F*, the oncosphere-forming ability was examined under microscope after the indicated treatments. Cells were magnified by 200 folds and the scale bar represented 5 μm. *G*, c-Myc and Cyclin D1 were determined by Western blot after the indicated treatments. β-actin was used as an internal control. *H*, the oncosphere-forming ability was examined under microscope after the indicated treatments. Cells were magnified by 200 folds and the scale bar represented 5 μm. CSC, cancer stem cell; HOTAIR, Hox transcript antisense intergenic RNA; NF-κB, nuclear factor-kappa B (∗ indicates *p* < 0.05; ∗∗ indicates *p* < 0.01; ∗∗∗ indicates *p* < 0.001.).

In order to study the signaling pathways that HOTAIR may participate in, we first extracted RNA from nontreated, HOTAIR knockdown and HOTAIR-overexpressed stem cells respectively, which were then used for real-time PCR detection in 96-well plate containing 90 key proteins in the stem cell–related pathways. The results showed that 13 RNA transcripts were aberrantly expressed in HOTAIR knockdown cells compared with HOTAIR-overexpressed cells (Fig. 4*A*). We then further verified the expression profiles of ALDH1A, c-Myc, Cyclin D1, Cyclin D2 by real-time PCR and found that their levels decreased significantly after HOTAIR knockdown while HOTAIR overexpression resulted in elevated expression of these genes (Fig. 4*B*), especially the expression of ALDH1A and c-Myc were consistent with the results we obtained earlier. Moreover, the expression of the four genes was further confirmed at the protein level (Fig. 4*C*). Based on literature search and GO/KEGG analysis, it was found c-Myc and Cyclin D1 are targets of the NF-κB signaling pathway (26, 27, 28). NF-κB was found to be involved in the maintenance of the CSC population in breast cancer (29, 30), but the precise mechanisms that how HOTAIR is involved in this pathway still needs to be further elucidated. To study whether HOTAIR is involved in the NF-κB signaling pathway to regulate stem cell self-renewal, we knocked down HOTAIR or inhibit NF-κB signaling pathway by PDTC and then examined the target protein expression. We found that the proteins c-Myc and Cyclin D1 were both decreased upon HOTAIR knockdown or PDTC treatment (Fig. 4*D*). Similarly, when p65 was depleted by shRNA, c-Myc and Cyclin D1 also decreased, the expression of which were also consistent with HOTAIR knockdown (Fig. 4*E*). Additionally, we also found HOTAIR knockdown or NF-κB interruption by shp65 or PDTC could abrogate oncosphere formation ability of these CSCs (Fig. 4*F*). These results indicated that HOTAIR and NF-κb signaling pathways could regulate c-Myc and Cyclin D1 expression respectively and were involved in the regulation of the stemness of breast cancer cells. In order to demonstrate whether HOTAIR correlated with NF-κb signaling pathway, we examined c-Myc and Cyclin D1 expression with or without NF-κB inhibition. The results showed that the expression of c-Myc and Cyclin D1 increased as expected upon HOTAIR overexpression; however, c-myc and Cyclin D1 level was antagonized by p65 knockdown or PDTC treatment upon HOTAIR overexpression (Fig. 4*G*). These results demonstrated that HOTAIR regulated the expression of c-Myc and Cyclin D1 through the NF-κB signaling pathway. Meanwhile, we also found NF-κB inhibition by PDTC abrogated sphere formation ability increased by HOTAIR overexpression (Fig. 4*H*). In summary, the results indicate that both HOTAIR and NF-κB pathway are necessary for c-Myc and Cyclin D1 expression and maintenance of the stemness.

**Figure 5:**
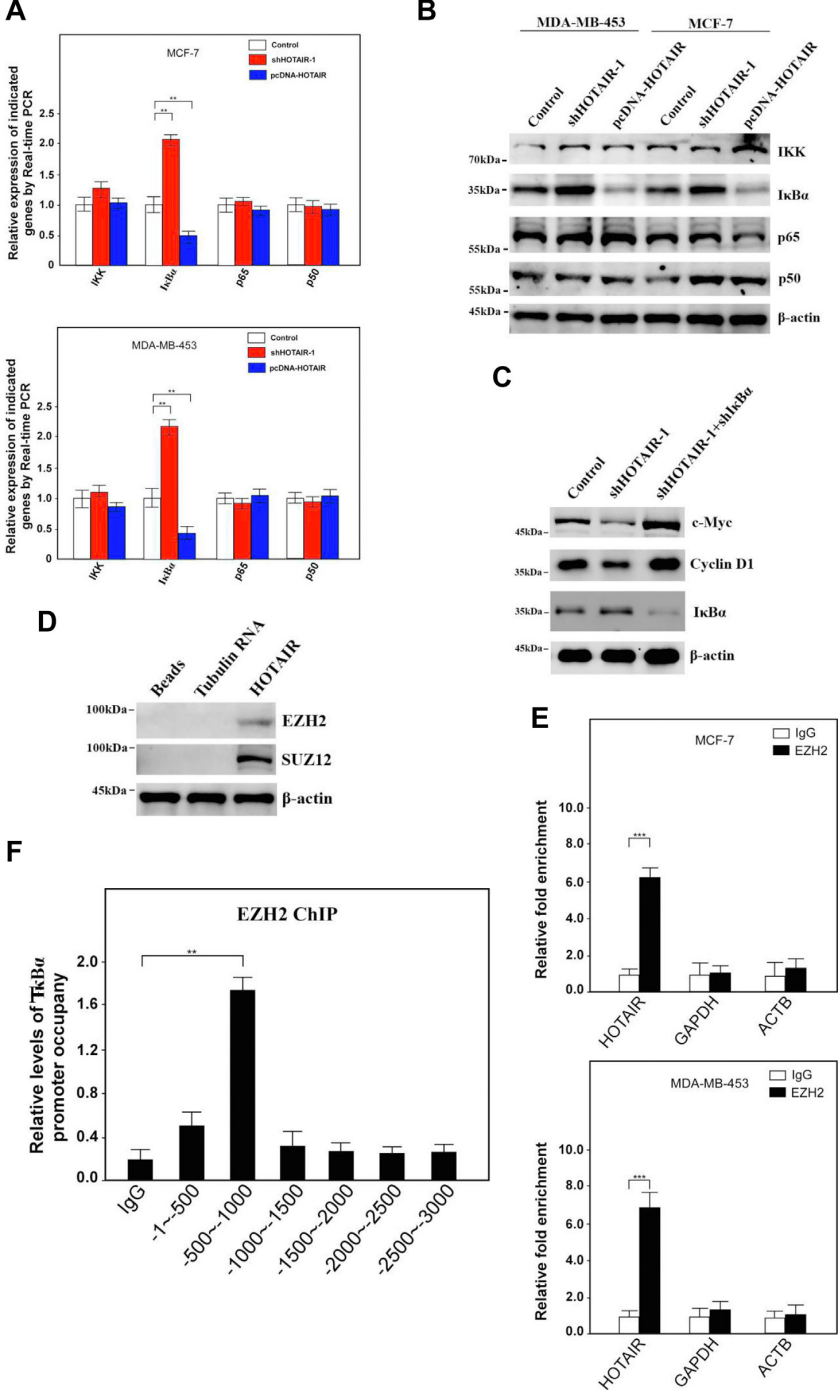

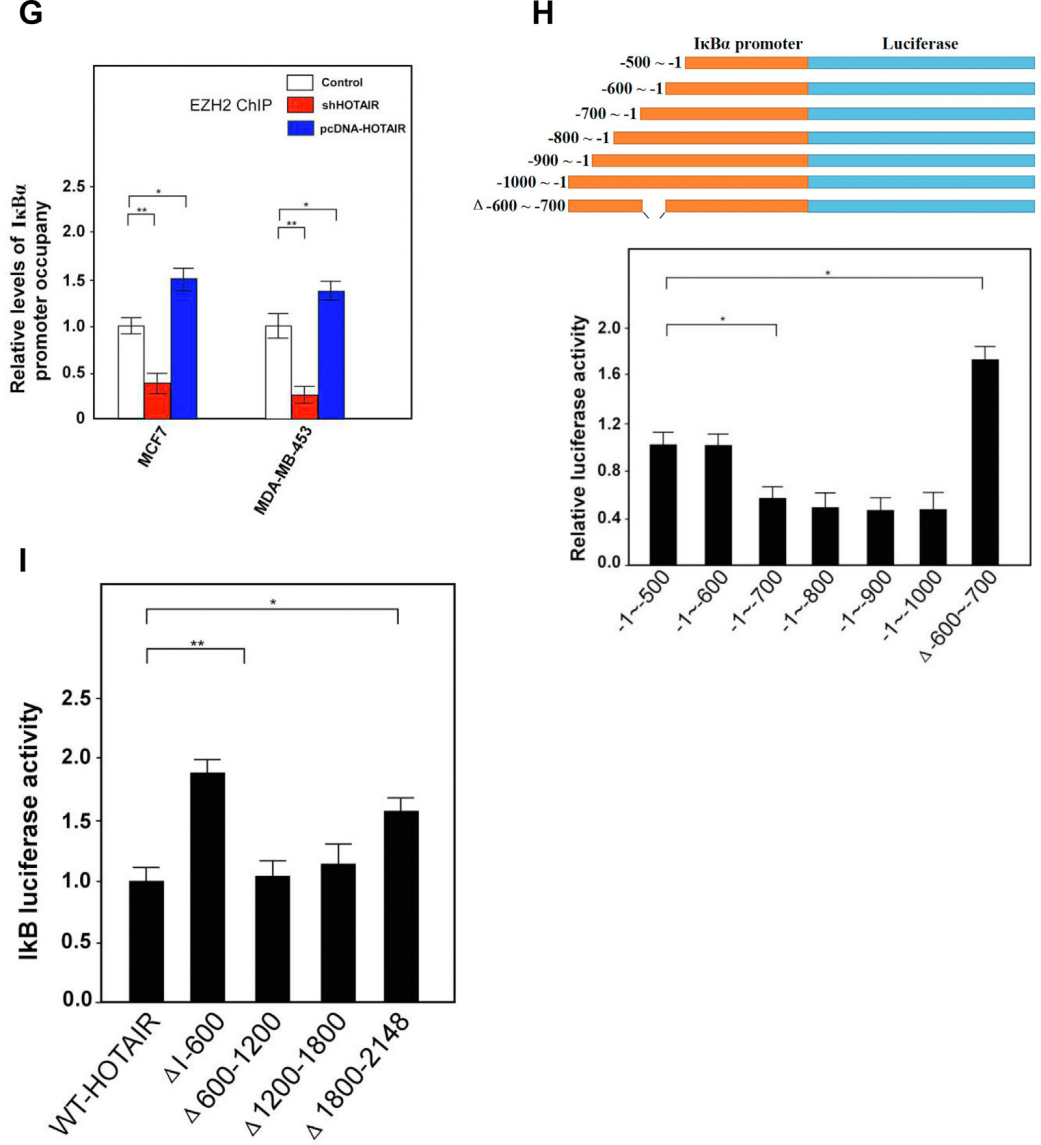
**HOTAIR recruits PRC2 complex onto IκBα promoter to inhibit the gene expression.***A*, the indicated genes were subjected to the expression analysis by real-time PCR after HOTAIR knockdown or overexpression. Relative gene expression was normalized to endogenous β-actin. Results are shown as means ± SD. *B*, the indicated genes were subjected to the expression analysis by Western blotting after HOTAIR knockdown or overexpression. β-actin was used as an internal control. *C*, the indicated genes were subjected to the expression analysis by Western blotting after shHOTAIR transfection alone or along with shIκBα. β-actin was used as an internal control. *D*, RNA pulldown assay was performed and two core components of the PRC2 were confirmed by immunoblotting. β-actin was used as a loading control. *E*, RNA immunoprecipitation (RIP) assay was performed and the indicated genes were subjected to real-time PCR. *F*, ChIP-quantitative PCR analysis was performed for the detection of EZH2-binding site in IκBα promoter. Data are shown as means ± SD. *G*, ChIP-quantitative PCR analysis was performed for the detection of EZH2-binding site in IκBα promoter after HOTAIR knockdown or overexpression. Data are shown as means ± SD. *H*, different loci of IκBα promoter were constructed into pGL4.18 vector (*upper panel*) and subjected to luciferase reporter assays (*lower panel*). Data are shown as means ± SD. *I*, pGL4.18- IκBα promoter reporter plasmid was transfected into cells with WT HOTAIR or truncated HOTAIR respectively and luciferase activity was measured. EZH2, enhancer of zeste homolog 2; HOTAIR, Hox transcript antisense intergenic RNA; PRC2, polycomb repressive complex 2 (∗ indicates *p* < 0.05; ∗∗ indicates *p* < 0.01; ∗∗∗ indicates *p* < 0.001.).

In order to further study how HOTAIR participated in the NF-κB signaling pathway, we first examined the expression profiles of key proteins in the pathway including p65, p50, IκBα, and IKK after HOTAIR knockdown or overexpression. Interestingly, when HOTAIR was overexpressed, IκBα decreased at both the mRNA and the protein levels (Fig. 5, *A* and *B*). On the contrary, upon HOTAIR knockdown, the expression of IκBα increased (Fig. 5, *A* and *B*). However, p65, p50, and IKK had not changed significantly regardless of HOTAIR knockdown or overexpression (Fig. 5, *A* and *B*). Moreover, the results showed that the expression of c-Myc and Cyclin D1 decreased upon knockdown of HOTAIR alone; however, while shIκBα was simultaneously transfected with shHOTAIR, the expression of c-Myc and Cyclin D1 recovered (Fig. 5*C*). The result preliminarily indicated that HOTAIR might participate in the regulation of the NF-κB signaling pathway by regulating the expression of IκBα. There has been reports that HOTAIR could recruit the PRC2 complex to regulate the expression of target genes (15, 25, 31, 32). In order to study whether HOTAIR could also interact with PRC2 in BCSCs, we carried out RNA pulldown and found that the components enhancer of zeste homolog 2 (EZH2) and suppressor of zeste 12 (SUZ12) of the PRC2 could be detected (Fig. 5*D*). Similarly, the interaction was further confirmed by EZH2 RIP assay (Fig. 5*E*). Therefore, we conducted ChIP assay to study whether HOTAIR could recruit PRC2 complex to regulate the expression of the IκBα and found that the EZH2 protein could bind to the promoter region of IκBα (Fig. 5*F*) and the main binding site was located in the 500bp-1000 bp upstream of the transcription start site. Upon knockdown of HOTAIR, EZH2 binding to the promoter region of IκBα was less efficiency while the promoter occupancy was increased by HOTAIR overexpression (Fig. 5*G*). Next, truncation or deletion of IκBα promoter were fused to luciferase reporters respectively to examine which region might be targeted by PRC2 complex. The results showed that luciferase activity significantly decreased as the truncation contained upstream sequences from −600 bp, however, as the deletion of −600 to −700 sequences in the promoter rescued luciferase activity (Fig. 5*H*). In order to determine which HOTAIR fragment was responsible to downregulate the IκB alpha expression, IκB alpha luciferase reporter plasmid was transfected into cells with WT HOTAIR or truncated HOTAIR respectively and luciferase activity was measured. The result showed that two fragments 1 to 600 and 1800 to 2148 were responsible for inhibition of IκB alpha as deletion of which would reduce the luciferase activity (Fig. 5*I*). In summary of the above data, HOTAIR could guide the PRC2 complex to the promoter region of IκBα, resulting in the regulation of this gene.

**Figure 6:**
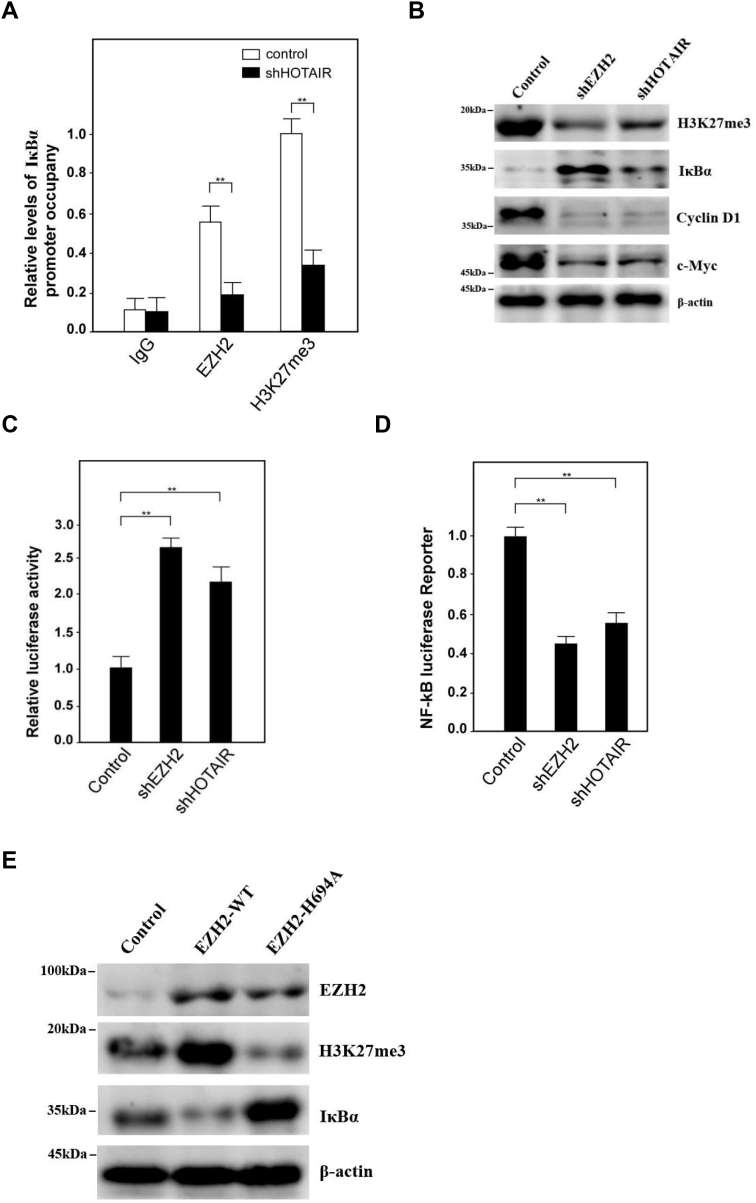

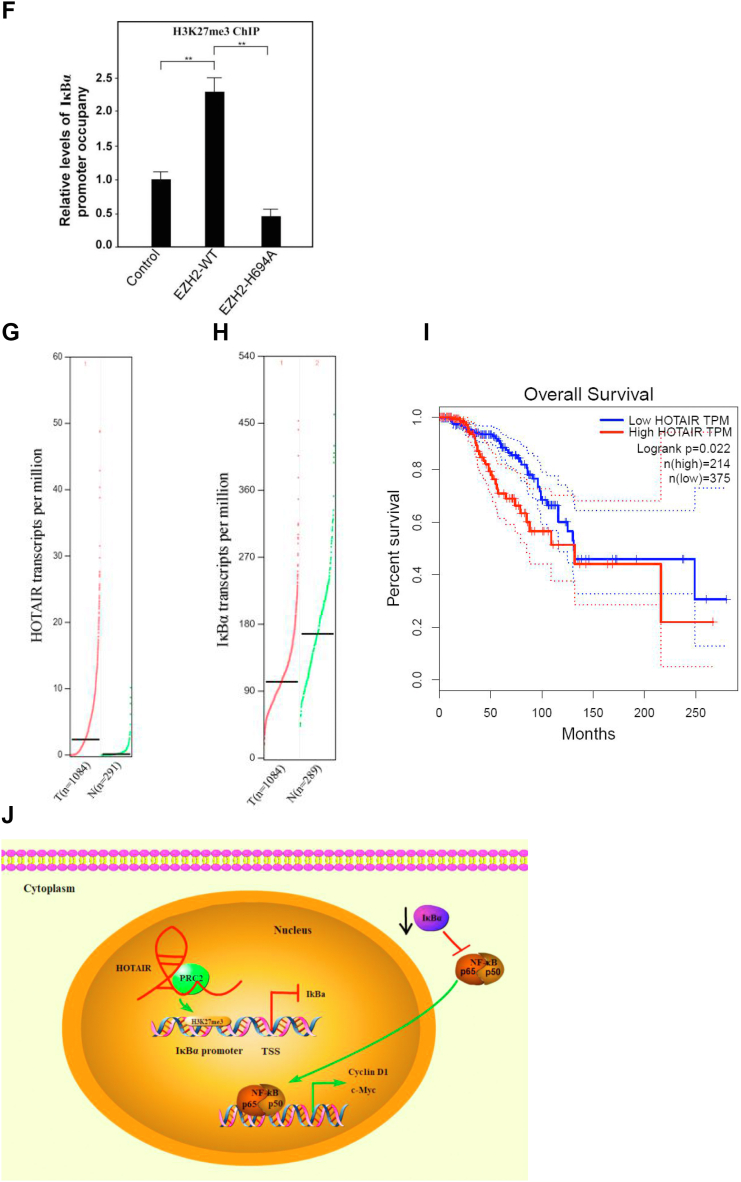
**HOTAIR recruits PRC2 complex onto IκBα promoter to inhibit the gene expression and negatively correlates with IκBα expression in clinical samples.***A*, ChIP-quantitative PCR analysis was performed for the detection of EZH2 and H3K27me3 binding site in IκBα promoter after HOTAIR knockdown. Data are shown as means±SD. *B*, the indicated genes were subjected to the expression analysis by Western blotting after EZH2 or HOTAIR knockdown. β-actin was used as an internal control. *C*, IκBα promoter were constructed into pGL4.18 vector and subjected to luciferase reporter assays after EZH2 or HOTAIR knockdown. *D*, NF-κB reporter plasmid was transfected into cells upon knockdown EZH2 or HOTAIR and luciferase activity was measured. *E*, the indicated genes were subjected to the expression analysis by Western blotting after EZH2-WT cDNA or EZH2-H694A cDNA transfection. β-actin was used as an internal control. *F*, ChIP-quantitative PCR analysis was performed for the detection of H3K27 histone triple-methylation–binding site within the IκBα promoter. Data are shown as means±SD. *G*, HOTAIR was detected in breast tumor tissues (n = 1084) and adjacent normal tissues (n = 291). The data was analyzed from GEPIA database. *H*, IκBα was detected in breast tumor tissues (n = 1084) and adjacent normal tissues (n = 289). The data was analyzed from GEPIA database. *I*, the survival rate of breast cancer patients with high or low HOTAIR expression was analyzed. *J*, a model describes how HOTAIR regulates the stemness of breast cancer cells. cDNA, complementary DNA; EZH2, enhancer of zeste homolog 2; H3K27me3, histone H3 lysine 27 trimethylation; HOTAIR, Hox transcript antisense intergenic RNA; NF-κB, nuclear factor-kappa B; PRC2, polycomb repressive complex 2 (∗∗ indicates *p* < 0.01.).

To further study how HOTAIR regulated IκBα expression, we found the PRC2 complex protein EZH2 as well as histone H3 lysine 27 trimethylation (H3K27me3) bound the IκBα promoter DNA with much less efficiency in HOTAIR knockdown cells compared with control cells (Fig. 6*A*). Additionally, H3K27me3 was decreased while IκBα was increased after EZH2 or HOTAIR knockdown, resulting in the reduction of both Cyclin D1 and c-Myc expression (Fig. 6*B*). A luciferase reporter assay also established that transcriptional activity of IκBα increased significantly in siEZH2- or siHOTAIR-treated cells compared with control cells (Fig. 6*C*). We then transfected NF-κB luciferase reporter plasmid into cells upon knockdown of EZH2 or HOTAIR and luciferase activity was measured. The result showed that upon knockdown of EZH2 or HOTAIR, NF-κB signaling pathway was inhibited (Fig. 6*D*). Moreover, we transiently cotransfected either WT or mutants (H694A, a SET domain mutant) EZH2 complementary DNA. Western blot assays showed that IκBα expression was reduced in EZH2-WT cells compared with empty vector-transfected cells. In contrast, overexpression of the methyl-transferase mutant EZH2-H694A increased IκBα expression while reducing H3K27me3 levels compared with EZH2-WT and vector control (Fig. 6*E*). Consistently, ChIP assays showed that EZH2-WT increased, but EZH2-H694A mutant reduced, H3K27 histone methylation within the IκBα promoter when compared with vector control (Fig. 6*F*). Next, we analyzed the expression of HOTAIR in clinical breast cancer patients from the GEPIA database, and the results showed that compared with normal breast tissue (N = 291), the expression of HOTAIR in breast cancer patients (N = 1084) increased significantly (Fig. 6*G*), indicating that HOTAIR was well correlated with breast cancer incidences, which was also consistent with the results we previously observed. However, in these samples, the expression of IκBα in breast cancers was significantly decreased, indicating IκBα expression was also negatively correlated with HOTAIR expression also in clinical samples (Fig. 6*H*). Finally, we analyzed the relationship between the survival rate of breast cancer patients and the expression profiles of HOTAIR. The results showed that the 10-years survival rate of patients with high HOTAIR expression was significantly lower than that of patients with relatively low HOTAIR (Fig. 6*I*). Collectively, these data provide evidence that H3K27 histone methylation is involved in silencing IκBα transcription and the IκBα level as well as the survival rate of patients with high HOTAIR expression was significantly lower than that of patients with relatively low HOTAIR.

In summary, HOTAIR could repress IkBα expression by recruiting PRC2 complex to the promoter of IkBα, resulting in the H3K27me3 and inhibition of IkBα expression. The decreased expression of IkBα was not able to inhibit NF-kB signaling pathway, promoting the expression of the downstream targets c-myc and Cyclin D1, the two of which were involved in the regulation of stemness of cancer cells (Fig. 6*J*).

## Discussion

Recently, CSCs have been identified in many solid tumors, including bladder, lung, breast, liver, colon, brain, and prostate cancers (33, 34, 35, 36, 37, 38, 39). CSCs are able to self-renewal and differentiation and might account for cancer relapse and metastasis due to their invasive and drug-resistant capacities (40). However, details of breast CSC biology remain for further investigation. Several markers including ALDH1 have been identified in BCSCs. In this study, we isolated the ALDH+ subpopulation of cells from breast cancer cell lines and identified them as BCSCs. Through real-time PCR analysis, we found HOTAIR was highly expressed in BCSCs and that HOTAIR played a critical role in maintaining the self-renewal of BCSCs.

LncRNAs have been reported to play widespread roles in gene regulation (12, 41), which exert their functions *via* diverse mechanisms, including modulation of gene expression, cotranscriptional regulation, cytoplasmic complexes, and pairing with other RNAs (14). Notably, increasing evidence indicates that lncRNAs modulate gene expression as epigenetic modifiers (16, 32, 42, 43, 44). The canonical pathway of NF-κB consists primarily of nuclear p65/p50 and is activated through the phosphorylation and following degradation of IκBα by IKKα/β. As a result, the degradation of IκBα can release the p65/p50 dimer to translocate from the cytoplasm into the nucleus and then stimulate the transcription of downstream target genes such as cyclin D1 and c-Myc (28). In this study, we found that HOTAIR inhibits IκBα expression in trans through recruitment of PRC2 onto the promoter, thus triggering the NF-κB signaling to initiate self-renewal of BCSCs. The NF-κB signaling plays a pivotal role in self-renewal and differentiation of CSCs. Aberrant activation of NF-κB signaling is implicated in breast cancer and other diseases (45, 46, 47). Interestingly, NF-κB was also found to regulate CSC populations in the basal-like breast cancer subtype in a non-cell–autonomous manner (47). Here, we show that depletion of HOTAIR and NF-κB subunit p65 impairs the self-renewal of BCSCs and their tumor-initiating capacity, suggesting that HOTAIR-mediated NF-κB signaling is critical for maintaining BCSC self-renewal and tumorigenicity. Self-renewal maintenance is a very complex biological process, which is regulated by pluripotent transcription factors, epigenetic states, and also many other cellular pathways (48, 49, 50). Our data support the idea that HOTAIR-mediated NF-κB signaling activation in BCSCs plays a critical role in the regulation of BCSC self-renewal. Furthermore, we also found that activation or inactivation NF-κB signaling could not affect the expression of HOTAIR (Fig. S2).

Cumulative data indicate that altered IκBα activity leading to cancers depends on NF-κB signaling pathway. In the ponasterone A-inducible IκBα SR mouse model, the induction of IκBα SuperRepressor delayed tumor appearance and reduced average tumor number. The induction of IκBα SuperRepressor in ponasterone A-inducible IκBα SR MEC lines *in vitro* could obtain the similar result (51). In this study, we demonstrate that IκBα inhibits the self-renewal of BCSCs and tumor propagation and acts as a tumor suppressor. IκBα is inhibited by HOTAIR in BCSCs to trigger the NF-κB signaling pathway, which primes the self-renewal of BCSCs. A recent study reported that IκBα functions as a tumor suppressor in tumorigenesis after ocular transplantation of embryonic stem cell–derived retinal progenitor cells (52). Consistently, our findings reveal that HOTAIR-mediated IκBα repression promotes breast tumorigenicity. Except for imposing constitutive activation of NF-κB, altered nuclear IκBα activity may either inhibit or potentiate PRC2-mediated regulation of stem cell– or differentiation-related transcription (53, 54, 55). CSCs are analogous to tissue-specific stem cells in that they contribute to long-term growth and are responsible for the maintenance and growth of tumors (56). Transcriptional regulation is one of the key regulatory mechanisms determining cell fate, which includes cis-regulatory elements and transacting factors. Among all transacting factors, chromatin remodeling regulation plays a pivotal role in gene transcription (57, 58). PRC2 has a histone methyltransferase activity and contains three core subunit, EZH2, SUZ12, and the embryonic ectoderm development (EED) (49). Furthermore, EZH2 is the main catalytic factor in PRC2 for H3K27me3, which usually indicates a status that chromatin is epigenetically repressed (59, 60). Emerging evidences indicate that EZH2 is indispensable for BCSC proliferation (61) and its specific silencing inhibits glioblastoma multiforme CSC self-renewal *in vitro* and tumor-initiating capacity *in vivo* (62). Here, we found that HOTAIR can recruit the PRC2 to inhibit IκBα expression, leading to priming of BCSC self-renewal.

In summary, HOTAIR can promote BCSC self-renewal and tumor propagation through activation of NF-κB signaling by recruiting the PRC2 complex to the IκBα promoter. Our findings reveal that HOTAIR represents an additional layer of regulation of BCSCs maintenance.

## Experimental procedures

### Cell culture and transfection

The cancer cell were maintained in Dulbecco’s modified Eagle’s medium (DMEM) (HyClone) supplemented with 10% fetal bovine serum (Gemini) and BCSC were maintained in DMEM/F12 with B27, EGF, and bFGF. All cells were incubated at 37 °C in a humidified chamber containing 5% CO2. Plasmids were transfected into cells with Lipofectamine 2000 (Invitrogen) according to the manufacturer’s instructions.

### Identification of ALDH+ cells by flow cytometry

Cells were washed with PBS at least twice and digested with 0.25% trypsin. Then, the obtained cells were washed with PBS three times. ALDH was labeled according to the manufacturer’s instruction. Briefly, 5 μl of the activated aldefluor reagent was added to 1.0 ml of the cell suspension. 0.5 ml of the mixture was immediately transferred to the control tube containing 5 μl of aldefluor deab reagent. Samples were incubated for 30 min at 37 °C. Following incubation, all tubes were centrifuged for 5 min at 250g and the supernatant was removed. Cell pellets were then resuspended in 0.5 ml of Aldefluor assay buffer and used for sorting the ALDH1+(high ALDH1 activity) and ALDH1- (low ALDH1 activity) cells by flow cytometry.

### Sphere formation assay

The breast cancer cells were seeded in ultra-low-attachment 6-well culture plates (Corning) in FBS-free DMEM medium supplemented with 2% B27 (Thermo Fisher Scientific) supplement without vitamin A, 20 ng/ml fibroblast growth factor (Sangon), 20 ng/ml epidermal growth factor (Sangon), 100 U/ml penicillin and 100 ng/ml streptomycin (Sangon), 4 μg/ml insulin (Sangon), and 20% methylcellulose (Sangon). Cells were incubated in a CO2 incubator for 1 week, and numbers of spheroid cells were counted under a microscope (Nikon).

### MTT assays

Cells were seeded (10^4^/well) in 96-well culture plates overnight. Then MTT solution (5 mg/ml) was added to each well and the cells were incubated at 37 °C for 4 h. Dimethyl sulfoxide was used to dissolve the formazan crystals, and a microplate reader (BioRad) was used to measure absorbance at 490 nm.

### Caspase-3/7 activities

The cells were washed twice with PBS after treatments, and 5 × 10^6^ cells were collected. Then, the cells were dissolved in 100 μl precooled lysis buffer and were shaken for 20 min, and the supernatant was collected for caspase-3/7 assay. The 50 μl supernatant was mixed with 50 μl of reaction buffer (2×). The cells were incubated under dark at 37 °C for 1 h. Luminescence was measured using SpectraMax M5(Molecular Device). Relative caspase-3/7 activity was obtained *via* calculation of the ratio to the reference standard.

### Lentivirus package

Lentiviruses were generated *via* transient transfection of 293T cells with lentiviral vectors expressing control or the gene of interest, cotransfected with the viral envelope vector pCMV-VSVG, and the viral packaging vector psPAX2. The medium was replaced 6 h posttransfection and lentiviral particles were concentrated by centrifugation at 10,000 rpm overnight and resuspended in PBS. Target cells were infected 24 h after plating.

### Generation of HOTAIR overexpression and knockdown cells

The sequences of HOTAIR were cloned using the primers F: 5′-AACCTCGAGCCAGTTCTCAGGCGAGAGCCGCGGC-3′ and R: 5′-ACGGGATCCATGCATAAAACCACCACACACACACA-3′ and inserted into the pLVX-IRES-ZsGreen vector. The two most effective shRNA sequences that targeting 5′-GAACGGGAGUACAGAGAGAUU-3′and 5′-CCACAUGAACGCC CAGAGAUU-3′ were selected and cloned into the pSilencer 2.1 vector. As a negative control, a nontarget scrambled sequence (5′-CTCCGATCGAGGATAATCGAA-3′) was also cloned into the same vector. The lentivirus generation and infection of target cells were the same as described previously.

### Quantitative real-time PCR

RNA was extracted from 2 × 10^6^ cells using TRIzol Reagent and 500 ng of total RNA was reverse-transcribed. The complementary DNA was used as the template under the following procedures: an predenaturation step at 95 °C for 3 min, followed by 40 cycles of denaturation and annealing/extension (95 °C for 5 s and 60 °C for 10 s). A melt curve (denaturation 95 °C for 5 s, annealing 65 °C for 10 s, and denaturation 95 °C for 5 s) was performed for specific amplification analysis. The primers for real-time PCR were listed in Table S1. All results were calculated using the 2^-ΔΔCt^ relative quantification method.

### Western blot

Cells were washed in PBS and subsequently lysed in RIPA lysis buffer (50 mM Tris–HCl, 150 mM NaCl, 0.1% SDS, 1% NP-40, 1× Protease inhibitor). Lysis was performed on ice for 30 min. Lysates were cleared by centrifugation at 8000*g* for 8 min, mixed with 1 × loading buffer (12% SDS, 30% Glycerol, 0.375 M Tris–HCl pH 6.8, 30% 2-mercaptoethanol, 1% bromophenol blue), and heated at 100 °C for 10 min. Subsequently, the proteins were separated on 8 to 10% SDS-PAGE gel and transferred onto the PVDF membranes (Millipore). Membranes were saturated and proteins were probed with primary antibodies and fluorescence-labeled secondary antibodies in 1× TBST buffer (1× TBS, 0.1% Tween). Then, the proteins were detected using a LI-COR Odyssey scanner and band intensities were quantified using LI-COR Image Studio Lite Version 3.1. The antibodies of c-Myc, SOX2, Oct4, Nanog, p50, EZH2 were purchased from Cell signaling Technology. The antibodies of CD44, CD24, Cyclin D1, ALDH1, p65, IKK, IκBα, SUZ12, H3K27Me3 were purchased from Abcam and β-actin was purchased from Santa Cruz.

### RNA pull-down assay

Three micrograms of biotinylated RNA were heated to 90 °C for 2 min, put on ice for 2 min, supplied with RNA structure buffer (10 mM Tris pH 7, 0.1 M KCl, 10 mM MgCl_2_), and then shifted to room temperature (RT) for 20 min to allow proper secondary structure formation. 10^7^ breast cancer cell pallets were resuspended in 2 ml PBS, 2 ml nuclear isolation buffer (1.28 M sucrose; 40 mM Tris–HCl pH 7.5; 20 mM MgCl_2_; 4% Triton X-100), and 6 ml water on ice for 20 min (with frequent mixing). Nuclei were pelleted by centrifugation at 2500*g* for 15 min. Nuclear pellet was resuspended in 1 ml RIPA buffer (150 mM KCl, 25 mM Tris pH 7.4, 0.5 mM DTT, 0.5% NP40, 1 mM PMSF, and protease Inhibitor (Roche Complete Protease Inhibitor Cocktail Tablets)). Resuspended nuclei were mechanically sheared using a dounce homogenizer with 15 to 20 strokes. Nuclear membrane and debris were pelleted by centrifugation at 13,000 rpm for 10 min. Folded RNA was then mixed with 1 mg of cell nuclear extract in RIPA buffer and incubated at RT for 1 hour. Sixty microliters of washed Streptavidin agarose beads (Sigma) were added to each binding reaction and further incubated at RT for 1 h. Beads were washed briefly five times in Handee spin columns (Pierce) and boiled in SDS buffer, and the retrieved protein was detected by standard Western blot technique.

### RNA immunoprecipitation

Cells at a concentration of 2 million cells/ml were treated with 0.3% formaldehyde in medium for 10 min at 37 ºC. 1.25 M glycine dissolved in PBS was added to a concentration of 0.125 M, and the sample was incubated for 5 min at room temperature. Cells were then washed twice in cold PBS and pelleted. The pellet was resuspended in 1 ml of RIPA buffer (50 mM Tris, pH 7.4, 150 mM NaCl, 1 mM EDTA, 0.1% SDS, 1% NP-40, and 0.5% sodium deoxycholate, 0.5 mM DTT and 1 mM PMSF/cocktail), incubated on ice with frequent vortex for 30 min, and the lysate was obtained by centrifugation at 13,000 rpm for 10 min. Antibodies were added and samples were incubated for 4 h at 4 °C. Samples were washed two times in RIPA buffer, four times in 1M RIPA buffer (50 mM Tris, pH 7.4, 1 M NaCl, 1 mM EDTA, 0.1% SDS, 1% NP-40, and 0.5% sodium deoxycholate), and then twice in RIPA in Handee spin columns (Pierce). The beads were resuspended in RIPA buffer and treated with proteinase K at 45 °C for 45 min. RNA samples were extracted with 1 ml Trizol. Proteins isolated before proteinase K treatment from the beads were detected by Western blot analysis. Coprecipitated RNAs were purified with RNeasy Mini Kit (Qiagen) and detected by qRT-PCR. Non-RT controls (without reverse transcriptase) were performed simultaneously to demonstrate that the signals were not from DNA contamination. The data of retrieved RNAs is calculated from the subtraction of RT/input ratio and non-RT/input ratio (for each experiment, n > 5).

### Chromatin immunoprecipitation and quantitative real-time PCR

Cells were crosslinked by addition of 1% formaldehyde and then the fixed cells were lysed in lysis buffer (140 mM NaCl, 50 mM Hepes–KOH pH 7.5, 1 mM EDTA, 1% Triton-X100, 0.1% SDS, 0.1% Na deoxycholate, and protease inhibitor cocktail). Then, the samples were subjected to sonication (10 times for 15 s at cycle 90% and output 40%) to shear the chromatin into fragments of 200 to 500 bp. Next, the lysate was precleared for 2 h with protein A/G beads and immunoprecipitated with corresponding antibody or normal IgG as isotype control. The immune complexes were then pulled down by protein A/G beads and washed once using RIPA wash buffer (150 mM NaCl, 50 mM Tris–HCl pH 8, 2 mM EDTA, 1% NP-40, 0.5% sodium deoxycholate, and 0.1% SDS) followed by three washes with wash buffer (150 mM NaCl, 20 mM Tris–HCl pH 8, 2 mM EDTA, 1% Triton-X100, and 0.1% SDS) and the last one wash with final wash buffer (500 mM NaCl, 20 mM Tris–HCl pH 8, 2 mM EDTA, 1% Triton-X100, and 0.1% SDS). The chromatin-antibody complexes were then eluted from the protein A/G beads using 1% SDS, 100 mM NaHCO_3_, and 10 mM DTT. Crosslinking was reversed with 5M NaCl for 6 h at 65 °C. Then the antibody-bound chromatin complexes were treated with RNAse for 1 h at 37 °C and proteinase K for 2 h at 45 °C. The DNA was extracted with phenol-chloroform, precipitated with ethanol, and dissolved in deionized water. Quantitative PCR reactions were performed to check the occupancy of corresponding proteins on promoters with primers by Real-time PCR.

### Luciferase reporter assays

Cells were transfected with pGL4.18 fused with the indicated promoters together with Renilla luciferase reporters expressing the renilla luciferase. After 48 h, renilla and firefly luciferase activities were measured by a Dual-Luciferase Reporter Assay System using a multifunctional microplate reader (Molecular Device). Renilla luciferase was used as an internal control.

### Xenograft tumor model

Animal experiments were performed according to the procedures approved by the Institutional Animal Care and Use Committee of Zhejiang University and their care was in accordance with institution guidelines. For the tumorigenesis assay, cells were injected subcutaneously into the left or right limb of 4-week-old male NOD/SCID nude mice. The mice were used for each cell clone and tumor size was measured every 3 days. The tumor volume was calculated as volume=(length/2) × width^2^. All mice were bred at pathogen-free conditions in the Animal Center.

### Clinical data extraction and TCGA analysis

Gene expression was analyzed in the website Gepia (http://gepia.cancer-pku.cn/). Differential expressions of HOTAIR and IκBα in breast tumor tissues and adjacent normal tissues were analyzed. The survival rate of breast cancer patients and the expression of HOTAIR was also analyzed in the website.

### Statistical analysis

Statistical analysis was performed using the GraphPad Prism 8. Statistical differences were analyzed by two-tailed Student’s *t* test or one-way ANOVA analysis. *p* values less than 0.05 were believed to be statistically significant.

## Data availability

All data that support the findings of this study are available from the corresponding authors upon reasonable request.

## Supporting information

This article contains supporting information.

## Conflict of interest

The authors declare no competing interests.
