# Supplementary material for: Long noncoding RNA HOTAIR regulates the stemness of breast cancer cells via activation of the NF-κB signaling pathway
Source: J Biol Chem. 2022 Oct 20;298(12):102630. doi: 10.1016/j.jbc.2022.102630 (PMC9691943; doi:10.1016/j.jbc.2022.102630)
Supplement: Supplementary Figure 1 [file mmc1.docx]

Supplementary Figure 1


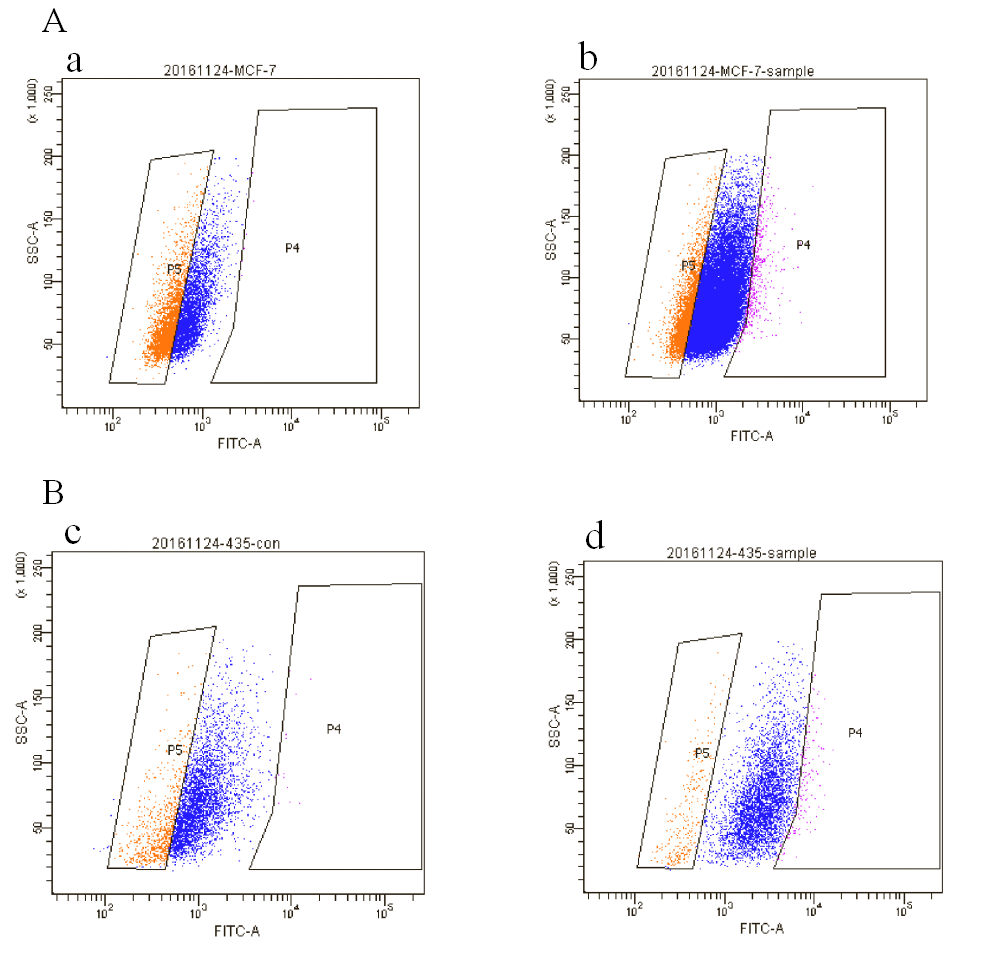


1. Flow cytometric sorting of ALDH1 positive and negative side population cells in MCF7. a: ALDH1 green fluorescence quenched control sample, b: ALDH1-labeled sample. (B) Flow cytometric sorting of ALDH1 positive and negative side population cells in MDA-MB-435. c: ALDH1 green fluorescence quenched control sample, d: ALDH1-labeled sample.
