# Supplementary material for: Long noncoding RNA HOTAIR regulates the stemness of breast cancer cells via activation of the NF-κB signaling pathway
Source: J Biol Chem. 2022 Oct 20;298(12):102630. doi: 10.1016/j.jbc.2022.102630 (PMC9691943; doi:10.1016/j.jbc.2022.102630)
Supplement: Supplementary Figure 2 [file mmc2.docx]

Supplementary Figure 2


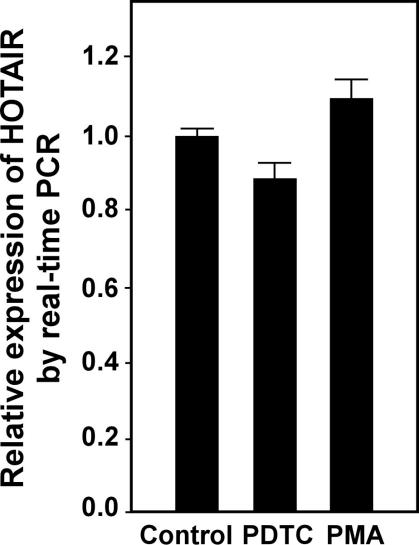


PDTC or PMA was used to inhibit or activate NF-κB signaling pathway and then cells were subjected to the analysis of HOTAIR by real-time PCR. Relative gene expression was normalized to endogenous β-actin. Results are shown as means±SD.
