# Supplementary material for: Long noncoding RNA HOTAIR regulates the stemness of breast cancer cells via activation of the NF-κB signaling pathway
Source: J Biol Chem. 2022 Oct 20;298(12):102630. doi: 10.1016/j.jbc.2022.102630 (PMC9691943; doi:10.1016/j.jbc.2022.102630)
Supplement: Supplementary Table 1 [file mmc3.docx]

|  | Forward Primers | Reverse Primers |
| --- | --- | --- |
| HOTAIR | CAAACAGAGTCCGTTCAGTGTC | AAAGGCTTTCCTATAACCCAAG |
| SOX2 | AAGTATCAGGAGTTGTCAAGGCAGAG | GTCCTAGTCTTAAAGAGGCAGCAA |
| c-Myc | AAGTATCAGGAGTTGTCAAGGCAGAG | GTCCTAGTCTTAAAGAGGCAGCAA |
| Oct4 | AGGTATTCAGCCAAACGACCATC | TCAGCTTCCTCCACCCACTTCT |
| Nanog | AGAATGAAATCTAAGAGGTGGCA | CCCTGGTGGTAGGAAGAGTAAA |
| ALDH1A | TTGATAAAGCCATAACAATCTCCTC | TCCAGACATCTTGAATCCACCA |
| Cyclin D1 | GTCCCACTCCTACGATACGCTAC | AACCAGCATCTCATAAACAGGTCA |
| Cyclin D2 | TTCTGGTATCTGGCGTTCTTTG | CAGGCTTGTCTGAGGAATGTTG |
| p65 | GACTACGACCTGAATGCTGTGCG | CGATTGTCAAAGATGGGATGAGAAAG |
| p50 | GGATTTCGTTTCCGTTATGTATG | CCTGAGGGTAAGACTTCTTGTTCT |
| IKKα: | GGCATGAGAAGATTAAGAAGAA | GTAAATGGCTACTAAACCGAAC |
| IκBα: | ATCCTGAAGGCTACCAACTACAATG | ATCAGCACCCAAGGACACCAAA |
| IκBα promter1-500 | TGTTTGGCCTGTGGTTGGAGAC | TTCCTCAACTGGCAATTAGGGT |
| IκBα promter 500-1000 | GTAATAAAGGCTTGAAGGGTCT | GGTTTCTTGTGACATTTGCTCC |
| IκBα promter 1000-1500 | ATTATCCCTTTCCTGATCCACA | AGACCACTAAATACTGGCTCCT |
| IκBα promter 1500-2000 | CTCAGCTCACTGCAACCTCCAC | GGCACCTGTAATCCCAGCTACT |
| IκBα promter 2000-2500 | GGTAAGATGGGAGTGGGGTGAT | TACTAAGGGCATCGTTCCAAAA |
| IκBα promter 2500-3000 | AATTCTCCTGCCTCAGCCTCCC | GGCGAAACCCCATCTCCACTAA |

Table 1 Primers used for Real-time PCR
